# Baduanjin Exercise, With or Without Vitamin D, Outperforms Vitamin D Alone in Reducing Frailty Among Institutionalized Older Adults: A Cluster-Based Randomized Controlled Trial

**DOI:** 10.3390/nu17233795

**Published:** 2025-12-03

**Authors:** Jing Gao, Ke Chen, Hui Xie, Ming Li, Si Chen

**Affiliations:** 1 School of Nursing and Rehabilitation, Shandong University, Jinan 250012, China; m15688873393@163.com (J.G.); cchenkee@163.com (K.C.); xiehui1122@mail.sdu.edu.cn (H.X.); liming74@sdu.edu.cn (M.L.); 2 Shandong Institute of Commerce and Technology, Jinan 250103, China

**Keywords:** Baduanjin, vitamin D, frailty, long-term care facilities, randomized controlled trial

## Abstract

**Background:** Frailty is a common condition among older adults residing in long-term care facilities and is associated with adverse health outcomes. Physical exercise and vitamin D supplementation have been proposed as preventive and therapeutic strategies, yet the combined effects of the two interventions remain uncertain. This cluster-based randomized controlled trial evaluated the independent and combined effects of Baduanjin exercise and vitamin D supplementation on frailty among residents of long-term care facilities. **Methods:** Sixty-four participants from three long-term care facilities in Jinan, China, were cluster-based randomized into a Baduanjin group (*n* = 23), a vitamin D group (*n* = 17), or a combined group (*n* = 24) for a 3-month intervention. The primary outcome was the frailty phenotype score, and the secondary outcome was serum 1,25(OH)_2_D_3_ concentration. Group comparisons were conducted using analysis of covariance (ANCOVA). **Results:** After the 3-month intervention, frailty phenotype scores were significantly lower in the Baduanjin (1.43 ± 0.90, *p* < 0.05) and combined (1.08 ± 1.10, *p* < 0.05) groups compared with the vitamin D group (2.65 ± 0.79), while no significant difference was observed between the Baduanjin and combined groups (*p* = 0.470). No significant between-group differences were found in the changes in 1,25(OH)_2_D_3_ levels (*p* = 0.215), whereas they were significantly increased from baseline in both the vitamin D (from 15.04 ± 2.67 to 18.43 ± 3.70, *p* < 0.05) and combined groups (from 14.77 ± 2.15 to 16.86 ± 2.50, *p* < 0.05). **Conclusions:** Baduanjin exercise, either alone or combined with vitamin D supplementation, effectively mitigated frailty among older adults residing in long-term care facilities.

## 1. Introduction

Frailty, a geriatric syndrome resulting from a decline in multisystem physiological reserves, has emerged as a major public health concern due to its high prevalence and associated adverse outcomes [1,2]. Epidemiological data indicate that frailty affects approximately 10–15% of community-dwelling older adults [3,4], while its prevalence in long-term care facilities reaches about 52.3%, several times higher [5]. This disparity highlights both the vulnerability of institutionalized older adults and the importance of targeted interventions within this population.

Exercise and nutrition represent the two most evidence-supported strategies for frailty management [6,7]. In terms of nutrition, vitamin D is a crucial protective factor, and its metabolic pathways are implicated in frailty pathogenesis [8]. Vitamin D influences skeletal muscle and physical performance through coordinated genomic and non-genomic mechanisms that regulate intracellular calcium homeostasis, mitochondrial bioenergetics, oxidative balance, and inflammatory signaling [9,10]. Zhou et al. [11] reported that individuals with serum 25(OH)D < 20 ng/mL were 2.7 times more likely to develop frailty. Notably, due to limited sun exposure, vitamin D deficiency affects over 80% of residents in long-term care facilities [12,13]. In terms of exercise, multicomponent exercise programs combining aerobic and resistance elements are well established for mitigating frailty but often require professional supervision and dedicated equipment, which limits participation among institutionalized older adults [14]. In contrast, Baduanjin, a traditional Chinese Qigong with a history of over a thousand years, integrates symmetrical physical postures, mindful focus, and breathing regulation into a harmonious practice. Comprising only eight simple movements grounded in Traditional Chinese Medicine theory, it is relatively accessible to learn and imposes minimal physical and cognitive demands [15,16]. Randomized controlled trials have shown that Baduanjin significantly improves physical and psychological outcomes in frail older adults, with high adherence and minimal risk [17,18].

Emerging evidence suggests that combining exercise with nutritional interventions may produce combined benefits for frailty [19]. Imaoka et al. [20] observed that neither exercise nor vitamin D supplementation alone reduced falls among frail older adults, whereas the combined intervention did. A meta-analysis further reported that vitamin D supplementation enhanced the effects of resistance exercise on muscle strength in healthy older adults, indicating a likely combined mechanistic link [21]. However, whether vitamin D supplementation can augment the effects of exercise on frailty improvement remains uncertain.

Therefore, this cluster-based randomized controlled trial aimed to evaluate the effects of Baduanjin, vitamin D supplementation, and their combination on reducing frailty and improving serum vitamin D status among older adults residing in long-term care facilities. The hypothesis is that the combined intervention of Baduanjin and vitamin D supplementation will be more effective in improving frailty in this population compared to either intervention alone.

## 2. Materials and Methods

### 2.1. Study Design

**Figure 1:**
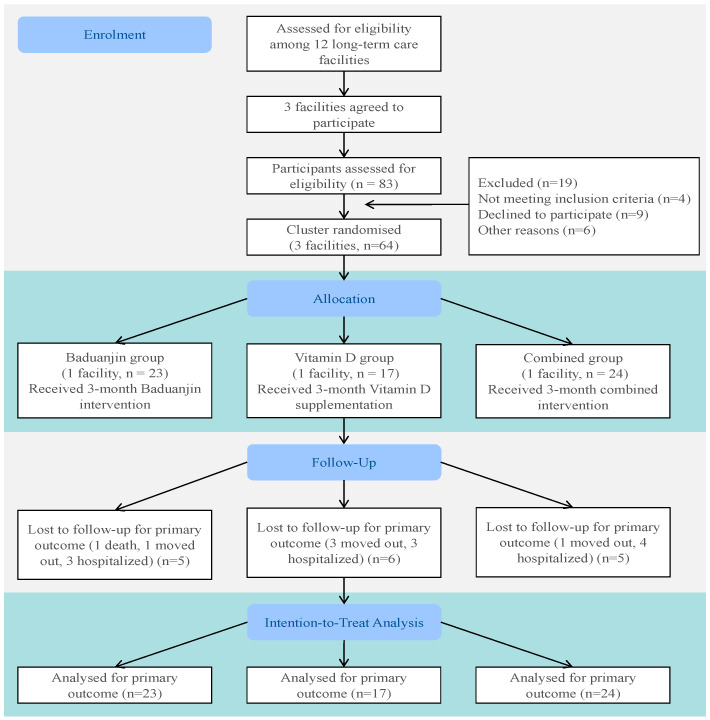
Flow diagram.

This study adopted a cluster-based randomized controlled trial design. Because the Baduanjin exercise could be easily observed and imitated within the same living environment, long-term care facilities were used as the unit of randomization to minimize cross-group contamination (Figure 1). The design and reporting of this trial were guided by the CONSORT 2010 extension for cluster randomized trials [22].

### 2.2. Participants

#### 2.2.1. Participant Recruitment

Of the initially contacted 12 long-term care facilities (four-star rating or higher) in Jinan, China, three facilities agreed to participate in the study. Participants were recruited through health education sessions held at the nursing facilities, where elderly residents were informed about frailty, the benefits of exercise, and the importance of nutrition. Booklets outlining the steps and precautions for Baduanjin were distributed to enhance participants’ understanding of the exercise.

Recruitment was carried out in accordance with predefined inclusion and exclusion criteria. Inclusion criteria were as follows: (1) age ≥65 years; (2) no visual or hearing impairment; (3) ability to walk and perform activities independently; and (4) willingness to participate and provide written informed consent. Exclusion criteria included (1) cognitive impairment; (2) diagnosed cardiovascular or cerebrovascular disease, severe renal disease, diabetic complications, or the presence of any cardiovascular symptoms or signs; and (3) participation in other intervention studies within the past 3 months.

#### 2.2.2. Sample Size Calculation

According to Lin et al. [23], the effect size of Baduanjin intervention on frailty ranges from 0.6 to 0.8 (average 0.7). Thus, power analysis was performed using G*Power 3.1, with a significance level of α = 0.05, power = 0.90, effect size = 0.7, numerator degrees of freedom = 2, and 3 covariates (baseline frailty score, sex, and age). The required sample size was estimated at 30 participants, and considering a 15% attrition rate, at least 36 participants were needed. In this study, among the 83 residents who met the inclusion criteria, 19 were excluded based on the above exclusion criteria, yielding a final sample of 64 participants enrolled in the study, meeting the sample size requirement. Ethical approval was obtained from the Ethics Committee of the School of Nursing and Rehabilitation, Shandong University (No. 2023-R-14). The trial was registered in the Chinese Clinical Trial Registry (ChiCTR2300069201).

### 2.3. Study Procedure

#### 2.3.1. Randomization and Blinding

Due to potential contamination among participants within the same facility, randomization was performed at the cluster level. The three participating facilities, therefore, served as three naturally formed clusters. The clusters were allocated in a 1:1:1 ratio to the Baduanjin group, the vitamin D group, or the combined group. Cluster allocation was conducted by an independent researcher, with no involvement in participant recruitment, intervention implementation, or outcome assessment. A randomization procedure was used to ensure the random assignment of each cluster to an intervention group. The final group sizes were as follows: Baduanjin group (*n* = 23), vitamin D group (*n* = 17), and combined group (*n* = 24). Outcome assessments before and after the intervention were conducted by non-facility staff members who were trained uniformly.

#### 2.3.2. Intervention Protocol

(1) Vitamin D Group: Participants received vitamin D supplementation based on their individual serum vitamin D levels and in accordance with the Chinese experts’ consensus on assessment and intervention for elderly patients with frailty [24]. At baseline, serum vitamin D levels were measured, and supplementation was adjusted in consultation with a physician according to the expert consensus. Most participants received two 400 IU capsules daily (800 IU total), while one participant received a reduced dose of one 400 IU capsule per day, adjusted to their baseline levels and medical needs. Supplementation was administered daily by caregivers after breakfast to ensure both adherence and safety. (2) Baduanjin Group: Training sessions were guided by certified Baduanjin instructors from the Shandong Qigong Center to ensure participants mastered the full set of movements. Group training was held three times per week, with each session consisting of two complete rounds of Baduanjin (approximately 30 min). Blood pressure and heart rate were measured prior to each session to ensure participants were fit to exercise safely, with any adverse readings prompting referral to medical staff. Adherence to group practice was monitored and recorded. (3) Combined Group: Participants in the combined group followed both Baduanjin exercises and vitamin D supplementation protocols. They received a daily dose of 800 IU of vitamin D, as determined by baseline serum vitamin D levels and medical needs, with no further adjustments required after initial testing.

The intervention lasted 3 months, a duration supported by evidence from previous randomized controlled trials. Systematic reviews indicate that mind-body exercises comparable to Baduanjin, such as Tai Chi, generally require at least 8–12 weeks to yield significant improvements in functional outcomes and reduce fear of falling among older adults [25]. Similarly, vitamin D supplementation generally requires approximately 2–3 months to achieve a new steady-state serum concentration [26].

### 2.4. Attendance

Intervention adherence was monitored and quantified for both the Baduanjin exercise and vitamin D supplementation. For the Baduanjin groups, adherence was calculated as the percentage of completed sessions out of the total prescribed sessions. The average adherence rates were 91% for the Baduanjin-alone group and 88% for the combined group. For vitamin D supplementation, adherence was defined as the percentage of days the supplement was taken as prescribed. The adherence rate was 100% for both the vitamin D alone and combined groups.

### 2.5. Measurement Indicators

#### 2.5.1. Primary Outcome

Frailty was objectively assessed using the Fried frailty phenotype, as recommended by the Chinese Medical Association for use in the Chinese population, including five components [24]: (1) Weight loss: unintentional loss of >4.5 kg or >5% of body weight in the past year. (2) Exhaustion: either of the following conditions occurred on 3 or more days during the past week: “I felt that everything I did was an effort” or “I could not get going”. (3) Weakness: grip strength was assessed on the self-reported dominant hand, with the highest of two trials recorded for analysis. Cutoffs were defined by sex and BMI. For males, weakness was defined as ≤29 kg for BMI ≤ 24.0 kg/m^2^, ≤30 kg for BMI 24.1–26.0 kg/m^2^ or 26.1–28.0 kg/m^2^, and ≤32 kg for BMI > 28.0 kg/m^2^. For females, the criteria were ≤17 kg for BMI ≤ 23.0 kg/m^2^, ≤17.3 kg for BMI 23.1–26.0 kg/m^2^, ≤18 kg for BMI 26.1–29.0 kg/m^2^, and ≤21 kg for BMI > 29.0 kg/m^2^. (4) Slowness: walking speed was assessed by measuring the time taken to complete a 4.57 m walk at the usual pace. Cutoffs were defined by sex and height. For males, slowness was defined as a time ≥7 s for those ≤173 cm in height, or ≥6 s for those >173 cm. For females, the criteria were a time ≥6 s for height >159 cm, or ≥7 s for height ≤159 cm. (5) Low physical activity: weekly energy expenditure, assessed by the International Physical Activity Questionnaire-Short Form, fell below the established sex-specific thresholds of 383 kcal for males and 270 kcal for females. Higher scores denote increased frailty, with a maximum of 5 points.

#### 2.5.2. Secondary Outcome

Serum 1,25(OH)_2_D_3_ concentration was determined using an enzyme-linked immunosorbent assay (ELISA). Dihydroxyvitamin D_3_ (DHVD3) ELISA kit (Catalog No.: CEA467Ge, 96T; Cloud-Clone Corp., Katy, TX, USA) was used for the measurement.

#### 2.5.3. Other Variables

Demographic and clinical characteristics (sex, age, medical history, medication use), body mass index (BMI), muscle mass, and body fat percentage (Model MC-980MA; TANITA, Tokyo, Japan). Adverse events were defined as any unfavorable or unintended sign, symptom, or disease temporally associated with the intervention. Serious adverse events were those resulting in death, hospitalization, or disability.

### 2.6. Statistical Analyses

Data analysis was performed using Stata 17.0. The intention-to-treat (ITT) principle was applied, and missing outcome data were handled by multiple imputation. Continuous variables are summarized as mean ± SD and median (IQR), and categorical variables as frequency and percentage. Normality was assessed using the Shapiro–Wilk test and homogeneity of variance was examined using Levene’s test. If both assumptions were met, one-way ANOVA was used for group comparisons; otherwise, the Kruskal–Wallis H test was applied. Within-group pre- and post-intervention comparisons were analyzed using paired-sample *t*-tests. Post-intervention between-group differences were assessed using analysis of covariance (ANCOVA), with baseline values, age, and gender as covariates. Partial η^2^ was used as an effect size measure, representing the proportion of total variance explained by the group after controlling for other variables. Given the cluster-randomized design, standard errors were adjusted for intracluster correlation using cluster-robust standard errors. Bonferroni correction was applied for multiple comparisons. A two-sided *p* value < 0.05 was considered statistically significant.

## 3. Results

### 3.1. Participant Characteristics

Table 1 presents the baseline characteristics of the participants. No significant differences were observed among the three groups in terms of sex, age, BMI, muscle mass, handgrip strength, or walking speed before the intervention (*p* > 0.05).

During the intervention period, no serious adverse events directly related to the intervention were observed. Mild muscle soreness or joint pain was reported by participants in the Baduanjin group (*n* = 4) and the combined group (*n* = 3), all of which resolved spontaneously within 2~3 days without medical treatment. One participant in the Baduanjin group died from respiratory failure secondary to influenza; after comprehensive assessment, the event was determined to be unrelated to the intervention. No intervention-related adverse events were reported in the vitamin D group.

### 3.2. Frailty Score Pre- and Post-Intervention Intervention

Table 2 summarizes changes in frailty scores before and after the intervention. Analysis of covariance (ANCOVA) was conducted using baseline frailty scores, age, and sex as covariates. Significant changes in frailty scores were observed between the groups (*p* = 0.002, partial η^2^ = 0.236). Between-group comparisons indicated that frailty scores were significantly lower in both the Baduanjin and the combined groups compared with the vitamin D group (*p* < 0.05). However, no significant difference was observed between the Baduanjin and combined groups (*p* = 0.470).

### 3.3. Vitamin D Concentrations Pre- and Post-Intervention

Table 3 shows the changes in serum 1,25(OH)_2_D_3_ levels before and after the intervention across the three groups. Between-group comparisons revealed no significant differences among the groups in serum 1,25(OH)_2_D_3_ changes (*p* = 0.215). Within-group analysis showed that serum 1,25(OH)_2_D_3_ levels remained unchanged in the Baduanjin group, whereas they were significantly increased in both the vitamin D and combined groups after the intervention (*p* < 0.05).

## 4. Discussion

This cluster-based randomized controlled trial examined the effects of three types of interventions—Baduanjin exercise, vitamin D supplementation, and their combination—on frailty among older adults residing in long-term care facilities. The findings showed that both Baduanjin and the combined interventions significantly improved frailty compared with vitamin D supplementation alone. These results contribute to the evidence base for the management of frailty in institutionalized older adults and may serve as a theoretical basis for developing more comprehensive clinical intervention strategies.

As a traditional Chinese Qigong, Baduanjin is characterized by gentle, coordinated movements and slow rhythms that make it particularly suitable for older adults with frailty. The present findings demonstrated significant improvements in frailty scores in both the Baduanjin and combined groups, consistent with previous studies. Liu et al. [17] reported that a 16-week Baduanjin intervention significantly enhanced handgrip strength and gait speed and reduced frailty scores by 1.3 points. Similarly, Tou et al. [18] confirmed the beneficial effects of Baduanjin across different frailty levels. A meta-analysis by Jones et al. [27] synthesizing 12 randomized controlled trials further supported that Baduanjin significantly improves lower-limb strength, balance, and quality of life among older adults.

Although vitamin D metabolic pathways have been suggested as one of the key physiological mechanisms associated with frailty [8], evidence regarding the efficacy of vitamin D supplementation in improving frailty remains inconclusive. In the current study, vitamin D supplementation demonstrated less improvement in frailty than either Baduanjin or the combined intervention, consistent with previous reports. Bolzetta et al. [28] found that, over 8 years of follow-up among 4421 older adults, monthly vitamin D intake was not associated with a reduced risk of frailty. Similarly, randomized controlled trials by Cai et al. [29] and Orkaby et al. [30] showed that even high-dose vitamin D supplementation did not prevent frailty. Other studies focusing on functional outcomes have also failed to demonstrate improvements in muscle strength or physical performance following vitamin D supplementation in frail older adults. For instance, Vaes et al. [31] reported no significant enhancement in muscle strength or function after vitamin D supplementation, and Hangelbroek et al. [32] found minimal or absent effects on muscle transcription factor expression, possibly because vitamin D receptors are predominantly present in proliferating satellite cells that respond mainly during muscle repair or exercise adaptation.

Notably, the present results indicated that the combined intervention yielded better improvement in frailty status compared with vitamin D supplementation alone, although the difference between the combined and Baduanjin groups was not statistically significant. This may partly be attributed to the relatively short intervention duration. Seino et al. [33] suggested that the effects of multidimensional interventions for frailty may require a longer exposure period to manifest fully. Imaoka et al. [20] also found that the combined use of exercise and vitamin D supplementation effectively reduced fall incidence among institutionalized older adults, but only after approximately 6 months of intervention. Baduanjin exercise primarily targets different aspects of frailty by improving physical function in key domains such as muscular strength, balance, and cardiorespiratory fitness [34]. In contrast, vitamin D influences skeletal muscle metabolism and systemic inflammation [35,36]. Simultaneously addressing these distinct pathways provides a mechanistic rationale for testing their combination in longer-term studies.

This study has several limitations. First, the absence of a no-intervention control group precludes the exclusion of Hawthorne effects or other non-specific factors as contributors to the observed outcomes. Second, baseline characteristics such as sex and age were imbalanced across groups, although adjusted for baseline frailty scores using ANCOVA, residual confounding may persist. Third, the 3-month duration might be too short to observe maximal changes in fundamental frailty components like muscle strength. Fourth, as the facility staff served as outcome assessors, potential bias cannot be ruled out. Finally, the use of serum 1,25(OH)_2_D_3_—a tightly regulated hormone with a short half-life [37]—instead of the stable 25(OH)D provides a physiological explanation for the high baseline variability and modest post-intervention rise, thereby limiting the interpretability of the vitamin D status data.

Future research should proceed in two directions. First, to directly address the methodological limitations identified in this study, larger-scale trials with a usual-care control group and a longer duration are needed, utilizing the standard 25(OH)D assay. Concurrently, the underlying biological mechanisms need to be elucidated. Future mechanistic studies should aim to define the specific pathways through which these interventions, individually and in combination, exert their effects. Evidence from the molecular level is therefore needed to refine interventions and characterize their combined effect.

The findings of this trial suggest practical relevance for long-term care. Baduanjin exercise represents a safe, low-cost, and easily implementable intervention that can be integrated into daily routines to mitigate frailty. For clinical practice, the results indicate that prioritizing structured physical activity like Baduanjin may yield more immediate improvements in frailty than vitamin D supplementation alone in this population. Although requiring verification, the trend favoring the combined approach suggests a holistic strategy that concurrently addresses physical performance and nutritional status. This integrated approach may be considered by healthcare providers and facility administrators seeking to optimize geriatric care.

## 5. Conclusions

This study integrated traditional Chinese Qigong Baduanjin with vitamin D supplementation, offering a safe, effective, and culturally appropriate intervention approach for frailty among older adults in long-term care facilities. The findings underscore that Baduanjin exercise, either alone or combined with vitamin D supplementation, effectively mitigated frailty among older adults residing in long-term care facilities. However, given the small sample size and the limited number of clusters, the results should be interpreted with caution. Future multicenter randomized trials with larger samples and longer follow-up are warranted to confirm these preliminary findings.

## Figures and Tables

**Table 1:** Baseline characteristics of the study participants.

Item	Total	Vitamin D Group	Baduanjin Group	Combined Group	*p*
	(*n* = 64)	(*n* = 17)	(*n* = 23)	(*n* = 24)	
Female, (%)	43.00 (67.19)	11.00 (64.71)	13.00 (56.52)	19.00 (79.17)	0.247
Age, (year)	81.50 ± 7.39	85.00 (82.00–89.00)	84.17 ± 7.57	80.67 ± 6.29	0.100
Disease history (number)	1.48 ± 0.78	1.35 ± 0.49	1.74 ± 0.81	1.33 ± 0.87	0.145
Multiple medications (type)	2.30 ± 1.00	2.29 ± 0.92	2.26 ± 1.01	2.33 ± 1.09	0.971
BMI (kg/m^2^)	23.72 ± 4.11	23.99 ± 3.23	23.07 ± 4.19	24.17 ± 4.63	0.634
Body fat percentage (%)	33.46 ± 9.57	36.03 ± 6.74	30.78 ± 10.68	34.56 ± 9.57	0.231
Muscle mass (kg)	37.07 ± 6.65	37.05 ± 6.78	38.30 ± 6.62	35.90 ± 6.68	0.488
Grip strength (kg)	16.11 ± 5.91	14.4 (10.60–15.55)	17.67 ± 7.15	15.97 ± 5.18	0.202
Walking speed (m/s)	0.63 ± 0.27	0.59 (0.36–0.78)	0.65 ± 0.27	0.63 ± 0.31	0.790

Note: Statistical analysis was conducted using either one-way ANOVA or Kruskal–Wallis H tests, depending on the data’s normality.

**Table 2:** Frailty score pre- and post-intervention in the three groups.

Group	Frailty Scores		
	Pre-Intervention	Post-Intervention	*p*
Vitamin D Group	3.47 (0.62)	2.65 (0.79)	0.002
Baduanjin Group	2.74 (0.75)	1.43 (0.90) ^a^	
Combined Group	2.88 (0.95)	1.08 (1.10) ^b^	

Note: Group differences post-intervention were analyzed using ANCOVA, adjusting for baseline values, age, and gender as covariates. Bonferroni correction was applied for multiple comparisons. ^a^: Baduanjin group vs. vitamin D group, *p* < 0.001; ^b^: combined group vs. vitamin D group, *p* < 0.05.

**Table 3:** Vitamin D concentrations pre- and post-intervention in the three groups.

Group	1,25(OH)_2_D_3_		
	Pre-Intervention	Post-Intervention	*p*
Vitamin D Group	15.04 (2.67)	18.43 (3.70) *	0.215
Baduanjin Group	20.12 (5.66)	19.08 (6.47)	
Combined Group	14.77 (2.15)	16.86 (2.50) *	

Note: Group differences post-intervention were analyzed using ANCOVA, adjusting for baseline values, age, and gender as covariates. Bonferroni correction was applied for multiple comparisons. Paired-sample *t*-tests were used for within-group comparisons of pre- and post-intervention data. * *p* < 0.05, comparison within the group pre- and post-intervention.

## Data Availability

The dataset is available upon reasonable request to the corresponding author. The data are not publicly available due to patient privacy.
